# USP22 supports the aggressive behavior of basal-like breast cancer by stimulating cellular respiration

**DOI:** 10.1186/s12964-023-01441-5

**Published:** 2024-02-12

**Authors:** Evangelos Prokakis, Husam Bamahmoud, Shaishavi Jansari, Lena Fritsche, Alexander Dietz, Angela Boshnakovska, Peter Rehling, Steven A. Johnsen, Julia Gallwas, Florian Wegwitz

**Affiliations:** 1 Department of Gynecology and Obstetrics, University Medical Center Göttingen, Göttingen, Germany; 2 Department of General, Visceral & Pediatric Surgery, University Medical Center Göttingen, Göttingen, Germany; 3 Institute of Pathology, University Medical Center Göttingen, Göttingen, Germany; 4 Department of Cellular Biochemistry, University Medical Center Göttingen, Göttingen, Germany; 5 The Robert Bosch Center for Tumor Diseases, Stuttgart, Germany

**Keywords:** Breast cancer, TNBC, Epigenetics, USP22, OXPHOS, CSCs, Therapy resistance

## Abstract

**Background:**

Breast cancer (BC) is the most frequent tumor entity in women worldwide with a high chance of therapeutic response in early- and non-metastatic disease stages. Among all BC subtypes, triple-negative BC (TNBC) is the most challenging cancer subtype lacking effective molecular targets due to the particular enrichment of cancer stem cells (CSCs), frequently leading to a chemoresistant phenotype and metastasis. The Ubiquitin Specific Peptidase 22 (USP22) is a deubiquitinase that has been frequently associated with a CSC-promoting function and intimately implicated in resistance to conventional therapies, tumor relapse, metastasis and overall poor survival in a broad range of cancer entities, including BC. To date, though, the role of USP22 in TNBC has been only superficially addressed.

**Methods:**

The current study utilized the MMTV-cre, *Usp22*^fl/fl^ transgenic mouse model to study the involvement of USP22 in the stem cell-like properties of the growing mammary tissue. Additionally, we combined high-throughput transcriptomic analyses with publicly available patient transcriptomic data and utilized TNBC culture models to decipher the functional role of USP22 in the CSC characteristics of this disease.

**Results:**

Interestingly, we identified that USP22 promotes CSC properties and drug tolerance by supporting the oxidative phosphorylation program, known to be largely responsible for the poor response to conventional therapies in this particularly aggressive BC subtype.

**Conclusions:**

This study suggests a novel tumor-supportive role of USP22 in sustaining cellular respiration to facilitate the drug-tolerant behavior of HER2^+^-BC and TNBC cells. Therefore, we posit USP22 as a promising therapeutic target to optimize standard therapies and combat the aggressiveness of these malignancies.

Video Abstract

**Supplementary Information:**

The online version contains supplementary material available at 10.1186/s12964-023-01441-5.

## Introduction

BC is a long-standing malady. In 2020, the world health organization (WHO) registered 2,261,419 newly diagnosed cases and 684,996 women (30.2%) ultimately succumbed to the disease worldwide [1]. In stark contrast to the beginning of the previous century when all women with diagnosed BC received an excessive surgical intervention as a uniform treatment, nowadays, the advent of targeted therapies has considerably revolutionized the current therapeutic strategies [2]. Indeed, the classification of BC into different subtypes according to the expression of estrogen receptor (ER), progesterone receptor (PR) and human epidermal growth factor receptor 2 (HER2) enabled the design of hormone deprivation or anti-HER2 agents that led to improved survival outcomes for these patients [3]. Unfortunately, in the case of TNBC, the lack of the expression of the abovementioned receptors renders this BC subtype impossible to treat with any available therapies other than conventional cytotoxic therapies [3]. Similarly, a great proportion of HER2^+^-BC patients fail to respond or rapidly develop resistance to anti-HER2 therapies due to compensatory tumor-promoting pathways, thereby leading to a frequent cancer relapse and metastasis [4]. Therefore, novel molecular targets with diagnostic and/or therapeutic potential are urgently needed.

During the last decades, accumulating evidence shows that TNBC is considerably enriched in CSCs which are characterized by aberrant self-renewal properties and with a unique capacity to develop and drive a chemoresistant phenotype [5]. Therefore, therapeutic targeting of CSCs, which are responsible for cancer cell repopulation, post-therapeutic relapse and metastatic dissemination, has drawn considerable research attention during the last years. In stark contrast to normal stem cells utilizing both glycolysis and OXPHOS as major metabolic programs for meeting their energy demands, CSCs develop a sustained OXPHOS-driven metabolic phenotype in a certain panel of cancer types such as ovarian, cervical, lung, pancreatic, breast cancer and glioblastoma [6, 7]. Indeed, augmented mitochondrial biogenesis was reported to be intimately associated with a more aggressive phenotype supporting the chemoresistant behavior of breast CSCs and ovarian cancer cells [8, 9]. In addition, a panel of inhibitors targeting the mitochondrial biogenesis transcriptomic program, the electron transport chain activity or the mitochondrial translation machinery, demonstrated anti-tumorigenic properties resulting in an efficient reduction of sphere-forming capacity (a characteristic property of CSCs) [10] derived from breast, ovarian, pancreatic, lung cancer, melanoma and glioblastoma entities [11]. In conclusion, mitochondrial OXPHOS activity represents a promising CSC-specific metabolic vulnerability for cancer patients suffering from frequent post-chemotherapeutic tumor relapses [12].

USP22 was initially identified as a member of a so-called "death-from-cancer" gene expression signature, comprising a group of genes whose expression strongly correlated with cancer aggressiveness, poor response to currently used therapies, tumor relapse and diminished patient survival [13]. The functional role of USP22 has been extensively studied by our group and others using several in vitro cell culture settings and genetically engineered mouse models [14]. As USP22 constitutes the deubiquitinating enzymatic module (DUBm) of the SAGA (Spt–Ada–Gcn5 Acetyltransferase) transcriptional co-activator complex, it has a major role in supporting transcriptional activation [14]. In the current work, we present a broad role of USP22 in supporting the OXPHOS-activity in several cancer entities including the most aggressive forms of BC (TNBC, HER2^+^-BC). Moreover, our findings demonstrate that USP22 epigenetically mediates the OXPHOS-dependent CSC- and drug-tolerant features, thereby, unveiling the potential therapeutic value of this DUB to combat the metabolic vulnerabilities of these particularly aggressive BC subtypes.

## Materials and methods

### Patient material, animal handling, mouse model generation and ethic approvals

Patient material has been collected in accordance with the ethical standards established in the 1964 Declaration of Helsinki and approved by the local ethical authorities (20/10/23). Animals were housed under specific pathogen-free (SPF) conditions and following the animal welfare laws and regulations of the state of Lower-Saxony (LAVES, registration number #15/1754).

### Mammosphere assay

A detailed protocol for mammary tissue dissection, mammary epithelial cell isolation, and primary cell culture from MMTV-cre; *Usp22*^wt/wt^ and MMTV-cre; *Usp22*^fl/fl^ tissues is provided in the Supplementary Data.

### Publicly available datasets

Publicly available patient transcriptomic and follow-up data were extracted from The Cancer Genome Atlas (source: https://portal.gdc.cancer.gov/; TCGA, retrieved from xenabrowser.net) to examine the association of *USP22, ATXN7L3, USP51* and *USP27X* expression with patient survival outcomes. Publicly available patient proteomic data derived from the Clinical Proteomic Tumor Analysis Consortium (CPTAC) and retrieved from cBioPortal (source: https://www.cbioportal.org/). ROC-based cancer patient response data to standard therapies were retrieved from ROC Plotter (source: https://www.rocplot.org/, TNBC-Affy id: 216964_at, HER2^+^-BC—Affy id: 220862_s_at). RFS and DMFS survival data were retrieved from KM-plotter (source: https://kmplot.com, Affy id: 200083_at) with the following patient cohort selection criteria: basal: PAM50-basal, IHC-score: ER-negative, PR-negative, HER2-negative; HER2^+^-BC: PAM50-HER2 + , IHC-score: ER-negative, PR-negative, HER2-positive.

Publicly available transcriptomic data based on USP22 deficiency were retrieved from Gene Expression Omnibus (source: https://www.ncbi.nlm.nih.gov/geo/) and ArrayExpress (source: https://www.ebi.ac.uk/biostudies/arrayexpress). Accession numbers of the utilized raw transcriptomic data are in detail mentioned in the respective figure legends.

### Cell culture, transfections, and functional assays

HCC1806 (ATCC® CRL-2335™), HCC1937 (ATCC® CRL-2336™), MDA-MB-231 (ATCC® HTB-26™), MDA-MB-468 (ATCC® HTB-132™), HCC1954 (ATCC® CRL-2338™) and SKBR3 (ATCC® HTB-30™) cells were purchased from the American Type Culture Collection (ATCC) and cultivated in RPMI 1640 GlutaMAX (Gibco; HCC1806, HCC1937, HCC1954), DMEM/F12 GlutaMAX (Gibco; SKBR3) or DMEM (Gibco; MDA-MB231, MDA-MB-468), supplemented with 10% fetal bovine serum (Sigma-Aldrich) and 1% penicillin/streptavidin (Gibco). Reverse siRNA transfections were performed using DharmaFECT 1 (Horizon Discovery) in OptiMEM GlutaMAX (Gibco) according to the manufacturer’s guidelines. A list of the siRNAs utilized in this study is provided in Table S1 of Supplementary data. Proliferation kinetics and tumorsphere numbers were recorded using a Celigo® S imaging cytometer (Nexcelom Bioscience LLC). Colony formation assays were stained with crystal violet and scanned with an Epson Perfection V700 Photo. Detailed protocols for siRNA transfection and functional assays can be found in Supplementary data.

### Immunofluorescence and immunohistochemical staining

*Immunofluorescence using mitotracker*: Cells were reverse-transfected with siRNAs in 6-well plates with coverslips and grown for another 72 h. Before cell fixation with 4% paraformaldehyde in PBS and washing with PBS, cells were cultured for 30 min with 350 nM mitotracker (M7512, Sigma Aldrich). Cells were permeabilized with 1% Triton X-100 in PBS for 10 min. Subsequently, coverslips were blocked for 1 h and incubated with the anti-USP22 primary antibody (Abcam, ab195289 at 1:200 dilution) overnight. Coverslips were washed with PBS-T (3x 10min) and a secondary antibody was applied with DAPI for 1 h at room temperature. Coverslips were washed with PBS-T (3x 10min) and mounted on microscope slides using mowiol. Analysis of mitochondrial morphology was performed on a mitochondrion-based approach using the MitochondriaAnalyzer plug-in [15] and based on the recommended guidelines (source: https://github.com/AhsenChaudhry/Mitochondria-Analyzer). Analysis of USP22 staining intensity was performed using ImageJ (v1.54 g). A detailed protocol for the respective analysis is provided at Supplementary Data.

*Hematoxylin and Eosin staining:* For hematoxylin and eosin (H&E) staining, nuclei were stained with hematoxylin solution (Carl Roth GmbH) for 1 min. Excess dye was removed using running tap water for 5 min. Counterstaining with eosin (Carl Roth GmbH) was performed for 5–10 min.

*Immunohistochemistry*: the detailed protocol is based on our previous work [16]. Briefly, 5 µm tissue sections were de-paraffinized in xylene and rehydrated using decreasing alcohol concentrations. Antigen retrieval and endogenous peroxidase block were performed in citric acid buffer (10 mM citric acid, pH 6, 0.1% Tween 20) and 3% H_2_O_2_ in PBS, respectively. Samples were then incubated in a blocking solution (5% bovine serum albumin (BSA, Merk) and 1% donkey serum (Dianova GmbH) in PBS). Anti-H2Bub1 antibody (mouse, homemade at 1:50 dilution) or anti-USP22 (Sigma-Aldrich, HPA044980 at 1:100 dilution) was diluted on blocking solution and samples were treated overnight. Samples were washed with PBS-T (3x 10 min) and secondary antibody (1:200, #711–065-150 or #711–065-152, Dianova) was diluted in blocking solution and incubated in a dark humidified chamber for 1 h. Samples were washed with PBS-T (3x 10 min) and ExtrAvidin-Peroxidase (Sigma-Aldrich) was diluted in PBS, and samples were incubated in a dark humid chamber for 90 min. Staining was developed using 3,3′-diaminobenzidine-tetrahydrochloride (DAB; Roth) and counterstained using hematoxylin. Slides were dehydrated following the reverse order of the alcohol gradient and mounted with Histokitt (Carl Roth GmbH).

### Fluorescence-Activated Cell Sorting (FACS)

After tryprsinization, 500,000 cells were washed twice with PBS and resuspended in 1 ml PBS/10% FBS (PBS/FCS, sterile filtrated). anti-CD44 (Biolegend, 103002) and anti-CD24 (biolegend, 311102) were added at 1:500 dilution and samples were incubated for 30 min in the dark at room temperature. Samples were centrifuged (300 g, 5 min) and washed twice with PBS/FCS and resuspended in 0.5 ml of PBS/FCS. Finally, CD44 and CD24 signal was measured using a FACS device (cytoflex, Beckman).

### Microscopy

IHC and H&E pictures from murine tissues were taken with a Zeiss Axio Scope A1. IHC pictures from patient material were taken with an Olympus IX83 microscope. Brightfield images of cultured cells were taken with a Nikon Eclipse S100 inverted microscope. Immunofluorescence pictures were acquired with an Olympus IX83 microscope.

### RNA isolation and real-time quantitative PCR (RT-qPCR)

RNA isolation, cDNA synthesis, and RT-qPCR were performed as previously described [17, 18]. Used primers for gene expression analysis are provided in Supplementary data in Table S2.

### mRNA sequencing data analysis

After verifying the RNA integrity on an agarose gel, mRNA sequencing (mRNA-seq) library was performed using the TruSeq RNA Library Prep Kit v2 Library. Library quality was assessed using an Agilent Bioanalyzer 2100. Paired-end (100 bp) sequencing was performed at BGI (Hong-Kong, China), using DNBSEQ™-G400 (BGI®). mRNA-seq data were then processed and analyzed in the Galaxy environment provided by the “Gesellschaft für Wissenschaftliche Datenverarbeitung mbH Göttingen” (GWDG). Briefly, the first 11 nucleotides of the raw reads were trimmed (FASTQ Trimmer). Human mRNA-seq data were aligned to the hg38 reference genome using the RNA STAR (version 2.4.0d-2). Read counts per gene were calculated with featureCounts (version 1.6.3 + galaxy2). Finally, differential gene expression analysis and normalized counts were obtained using DESeq2 (version 2.11.40.6 + galaxy1). To identify differentially regulated genes upon USP22 loss, we used a cut-off of |log2 fold change|≥ 0.7; p-val < 0.05 and basemean ≥ 15. Pathway enrichment analysis was performed using the Gene Set Enrichment Analysis (GSEA, v4.3.2, source: https://www.gsea-msigdb.org/gsea/msigdb). Raw sequencing data are accessible at ArrayExpress (https://www.ebi.ac.uk/arrayexpress/) with the following ArrayExpress accession number: E-MTAB-13577. The accession number of other used publicly available NGS data is stated in the Supplementary Data.

### ChIP sequencing data analysis

Processing of sequenced data was performed in the Galaxy environment (galaxy.gwdg.de). Briefly, ChIP-seq reads were mapped to the hg38 reference genome assembly using Bowtie2 (version 2.3.2.2). PCR duplicates were removed using the RmDup tool (version 2.0.1). The deeptools suite (version 3.2.0.0.1) was utilized to generate normalized coverage files (bamCoverage), call peak changes (bigwigCompare), and generate aggregate plots and heatmaps (computeMatrix and plotHeatmap).

### Oxygen consumption rate measurement

Oxygen Consumption Rate (OCR) was measured with a Seahorse XF Analyzer (Agilent) according to the manufacturer’s recommendations. Briefly, 60.000 cells per well were seeded one day before the measurement. Before OCR measurement, cells were washed with PBS and maintained in 180 ul of XF assay medium in a non-CO_2_ incubator for 45 min. Meanwhile, the cartridge was loaded with Oligomycin (30 µM), FCCP (15 µM), Antimycin (10 µM), Rotenone (10 µM), Glucose (25 µM) and 2-DG (25 µM). The OCR measurement was performed running the standard preset mito stress test program. OCR values were normalized to the quantified protein amount of each well using the CyQUANT kit and based on manufacturer’s instructions (Thermofisher Scientific). Results were graphed using GraphPad Prism v8.0.1.

### Statistical analysis of experimental findings

All used statistical tools are in detail stated for each experiment at the respective figure legends.

## Results

### Mammary-specific loss of *Usp22* impairs the stem cell-like properties of the growing murine mammary gland

**Fig. 1:**
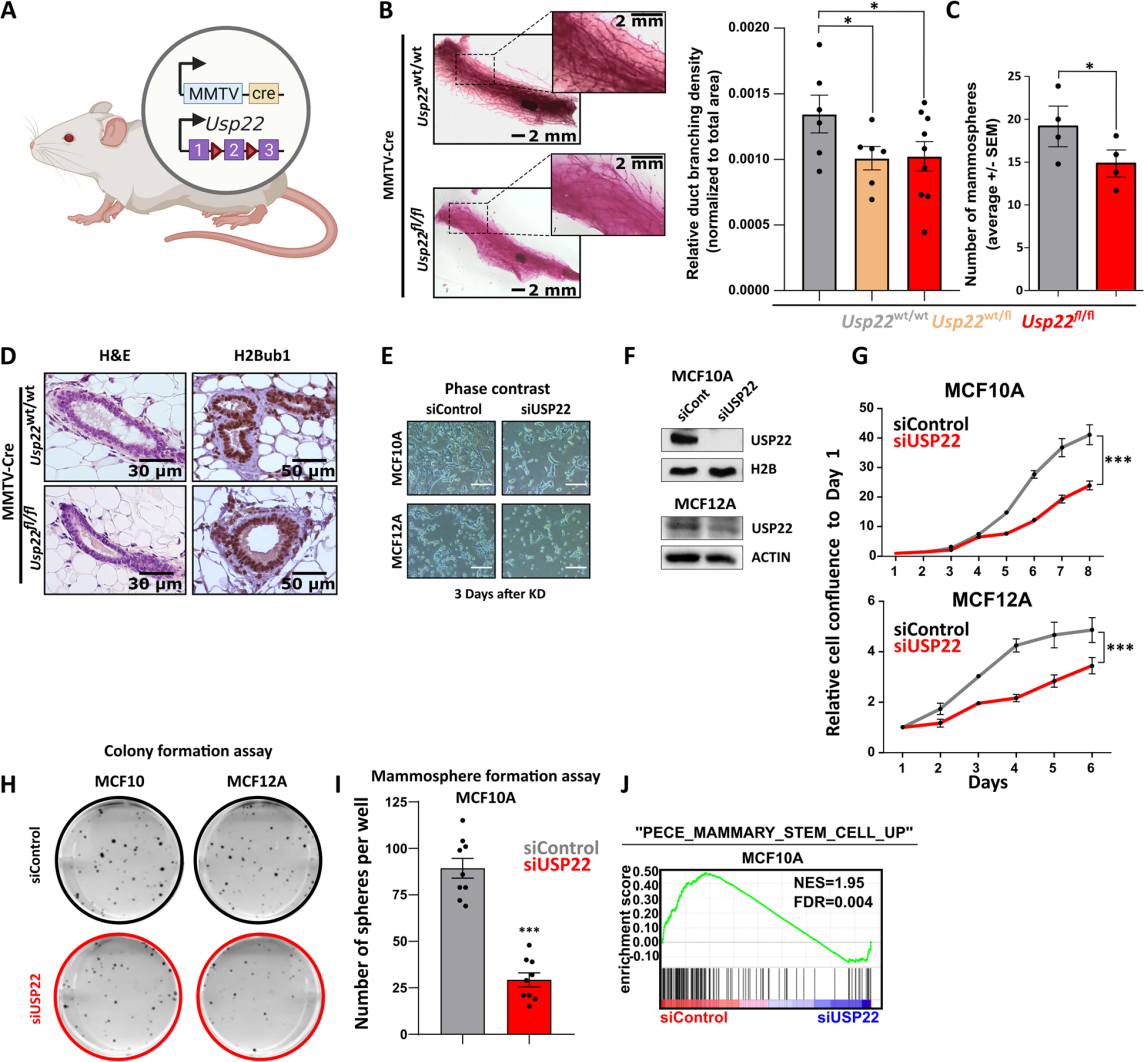
Mammary tissue-specific loss of *Usp22* impairs the stem cell-like properties of the growing murine mammary gland: **A** Schematic representation of the two transgenes of the MMTV-cre; *Usp22*^fl/fl^ mouse model. **B** Whole mounts staining of mammary glands showing a significant decrease of mammary duct branching density in MMTV-cre; *Usp22*^fl/fl^ mice compared to the control group with representative brightfield pictures (left panel) and the respective quantification of branching density and number of ex vivo cultured mammospheres (right panel). **C** Mammosphere formation assay mammary epithelial cells from MMTV; *Usp22*^wt/wt^ and MMTV; *Usp22*^fl/fl^ mice. **D** Hematoxylin and eosin staining (left panel) and immunohistochemical detection of H2Bub1 (right panel) on mammary gland sections from MMTV-cre; *Usp22*^fl/fl^ mice and MMTV-cre mice. **E-I** Brightfield pictures (white scale bar: 100 µm) (**E**), western blot analysis of USP22 protein (**F**), growth kinetics (**G**), colony formation assay (**H**) and mammosphere formation assay (**I**) in siControl- and siUSP22-treated MCF10A and MCF12A cells. **J** Gene set enrichment analysis (GSEA) performed on the high-throughput RNA sequencing data of siControl- and siUSP22-treated MCF10A cells (accession number: E-MTAB-8247). Statistics: B (right panel): One-way Anova; C, G (based on the area under the curve = AUC); I: Student t-test. *p<0.05, ***p<0.005. All experiments were performed in at least three biological replicates

In previous work, we combined the HER2-driven BC mouse model with a mammary-specific loss of *Usp22* showing that this DUB exerts a profound pro-tumorigenic role in this particular aggressive malignancy [16]. However, the in vivo impact of mammary-specific loss of *Usp22* on the stem cell properties of the growing mammary gland, which are important features of the aggressive behavior of TNBC, had not been explored to date. To shed light on this direction, we crossed MMTV-Cre mice harboring a mammary tissue-specific expression of the Cre recombinase with mice harboring a loxP-flanked *Usp22* allele (Fig. 1A). Indeed, *Usp22*^wt/fl^ as well as *Usp22*^fl/fl^ mice showed a reduced mammary duct branching density (Fig. 1B) compared to their control counterparts, a finding consistent with reduced mammary stem cell properties [19, 20]. Noteworthy, this observation strongly aligns with a recently published study showing an increase of these stem cell features in USP22-overexpressing murine mammary tissues, reinforcing the validity of our findings [21]. Moreover, a significant reduction in mammosphere formation was observed in *Usp22*-deficient mammary epithelial cells compared to wild-type counterparts, a parameter consistent with impaired stem cell features (Fig. 1C) [20, 22]. Microscopic examination of wild-type and *Usp22*^fl/fl^-derived mammary tissues did not reveal any observable histomorphological differences (Fig. 1D, left panel). In addition, monoubiquitination of histone 2B at K120 (H2Bub1), a deubiquitination target of USP22 [23], showed no difference in mammary epithelial structures lacking *Usp22* compared to wild-type mice (Fig. 1D, right panel), presumably due to other compensatory H2Bub1-specific DUBs [23]. To confirm its stemness-promoting role, we silenced USP22 in two normal human mammary epithelial cell lines (MCF10A and MCF12A (Fig. 1E). As expected, after successful silencing of USP22 in both cell lines (Fig. 1F), we confirmed that USP22-depleted cells showed an impaired growth kinetic, clonogenic capacity and sphere formation potential (Fig. 1G-I). Strikingly, mRNA-sequencing (mRNA-seq) in MCF10A cells showed that USP22-silenced MCF10A cells significantly reduced the mammary stem cell-specific transcriptomic signature (Fig. 1J). Therefore, our results demonstrate that USP22 fosters the stemness capacity of mammary epithelial cells in vivo and in vitro.

### USP22 is related to CSC properties and an unfavorable prognostic outcome in BLBC patients

**Fig. 2:**
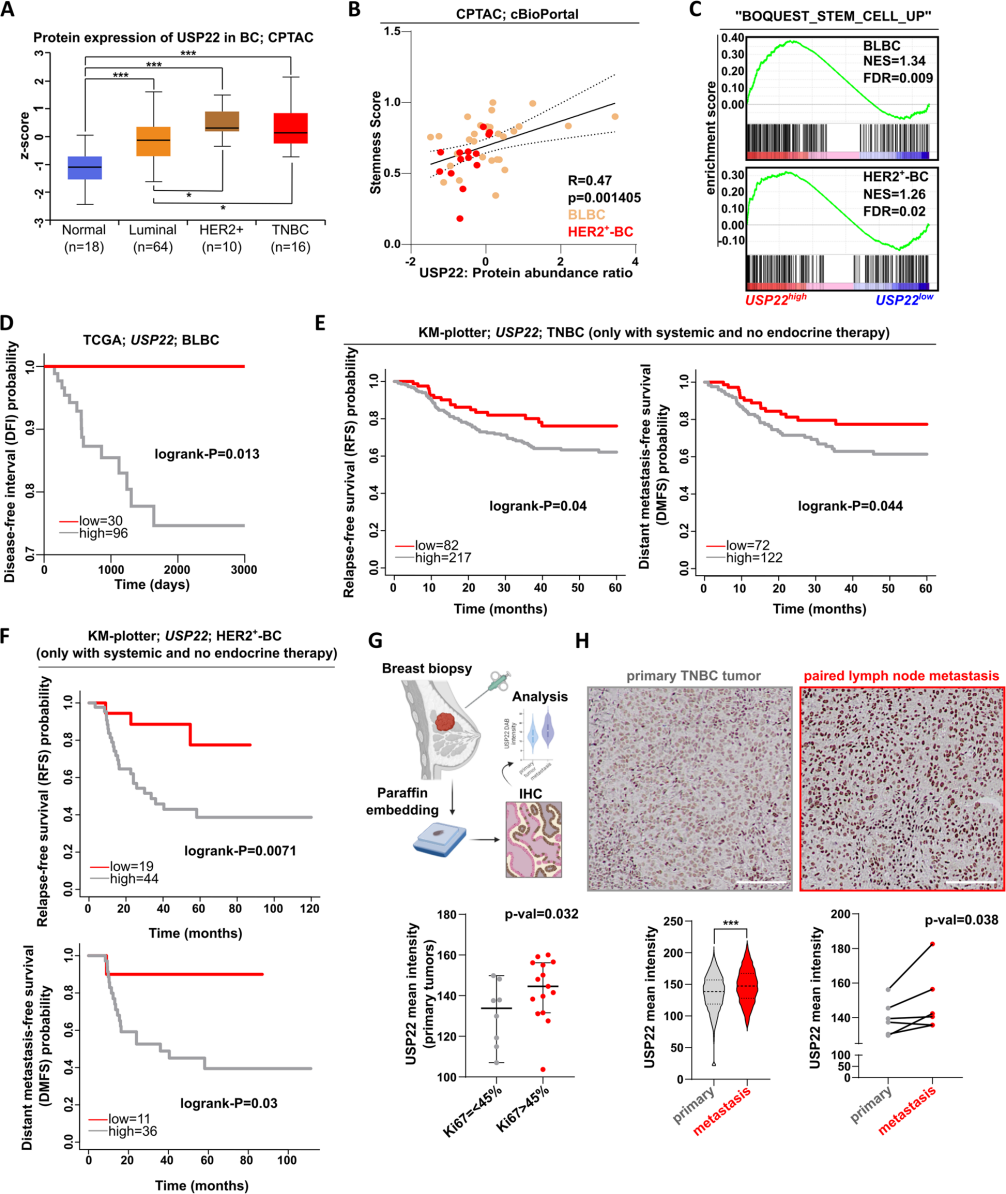
USP22 is related to CSC properties and an unfavorable prognostic outcome in TNBC patients:** A** USP22 protein levels in healthy mammary tissue and different BC subtype biopsies (source: Clinical Proteomic Tumor Analysis Consortium-CPTAC, retrieved from: http://ualcan.path.uab.edu/). **B** Correlation of USP22 protein expression with the stemness score in several TNBC and HER2^+^-BC biopsies (source: Clinical Proteomic Tumor Analysis Consortium-CPTAC, retrieved from: https://www.cbioportal.org/). **C** GSEA of the high-throughput RNA sequencing data from basal-like BC (BLBC) and HER2^+^-BC patients strongly enrich the "BOQUEST_STEM_CELL_UP" gene signature in *USP22*^high^-expressing patients. NES: Normalized enrichment score. **D** *USP22* expression significantly correlates with a poor survival outcome in BLBC patients (source for **C**-**D**: https://portal.gdc.cancer.gov/). **E–F** Patient survival analysis indicates a high probability of disease recurrence in TNBC (**E**) and HER2^+^-BC patients (**F**) with high expression of *USP22* (source: https://www.rocplot.org/). **G-H **Immunohistochemical detection of USP22 in treatment-naive primary (*n* = 23) and lymph node metastasis (*n* = 8) biopsies showed an increase of USP22 levels in Ki67^high^ compared to the Ki67^low^ primary tumors (**G**, lower panel). In addition, USP22 expression was particularly increased in lymph node metastases compared to primary tumors (**H**, upper panel), as well as in paired metastasis biopsies compared to their primary tumor counterparts (**H**, lower panel). White scale bar: 100 µm. Statistics: B: Pearson correlation analysis; G: Student t-test; H (lower-left panel): non-parametric Mann–Whitney test; H (lower-right panel): paired t-test. ***p<0.005

Until now, we demonstrated that USP22 supports the stem cell-like properties of mammary epithelial cells. To translate these findings in TNBC, which is particularly enriched in CSCs, we initially analyzed publicly available patient data. Hence, we assessed USP22 protein expression in various BC subtype lesions and normal breast tissue specimens. USP22 levels were found higher in cancerous lesions compared to normal tissues, independently of the BC subtype, and particularly high in TNBC biopsies (Fig. 2A). Furthermore, we identified that USP22 mRNA and protein levels in TNBC and HER2^+^-BC biopsies, the two most challenging BC subtypes, are highly correlated with a stemness signature (Fig. 2B-C) and poor survival outcome (Fig. 2D-F). To further consolidate the prognostic value of USP22, we performed immunohistochemical detection of this marker in treatment-naive primary tumor (*n* = 23) and treatment-naive lymph node metastases (*n* = 8) biopsies collected from 23 TNBC patients. As expected, USP22 expression was associated with the Ki67 proliferation index marker within the group of primary tumor biopsies (Fig. 2G) and was increasingly expressed in all lymph node metastases (Fig. 2H), strongly pointing to a profound role of this DUB in the progression and metastatic dissemination of this disease.

### USP22 supports the tumorigenic properties of TNBC cells

**Fig. 3:**
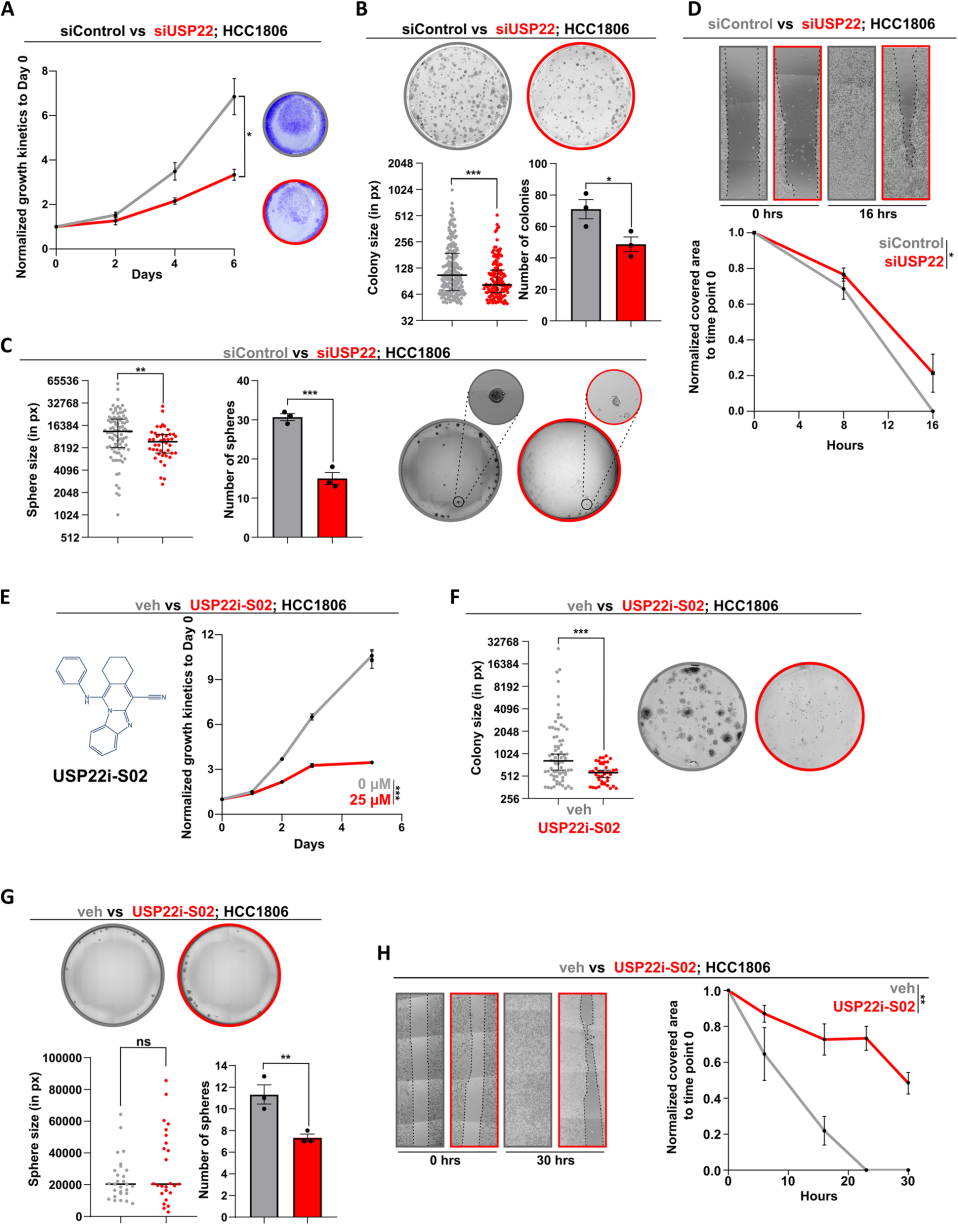
USP22 supports the tumorigenic properties of TNBC cells. **A**-**D** Growth kinetics (**A**), colony formation assay (**B**), tumorsphere formation assay (**C**) and gap closure assay (**D**) strongly point to a loss of the tumorigenic properties upon USP22 silencing in TNBC cells. **E–H** Growth kinetics (**E**), colony formation assay (**F**, 25 µM), tumorsphere formation (**G**) and gap closure assay (**H**, 50 µM) of DMSO (veh)- and USP22i-S02-treated TNBC cells. Statistics: **A**, **D**, **E** and **H** (based on the area under the curve = AUC), B-C, G right panels: Student t-test; B-C, F-G left panels: non parametric Mann Whitney test; *p<0.05, **p<0.01, ***p<0.005. All experiments were performed in at least three biological replicates

We leveraged several TNBC cell lines to study the consequence of USP22 loss in vitro using a smart pool of single USP22-specific siRNAs (Fig. S1A). Consistent with a CSC-associated role in TNBC (Fig. 2B-C) and a MaSC capacity in murine mammary tissues (Fig. 1), USP22 depletion impaired the cell growth (Fig. 3A, Fig.S1B), colony (Fig. 3B, Fig. S1C), tumorsphere formation capacity (Fig. 3C, Fig. S1D) and migratory behavior (Fig. 3D, Fig. S2A) in TNBC cell lines. In addition, pharmacological inhibition of USP22 using the USP22i-S02 compound effectively abrogated the growth capacity (Fig. 3E, Fig. S1E), clonogenic potential (Fig. 3F, Fig. S1F), tumor sphere formation capacity (Fig. 3G, Fig. S1G) and migratory behavior (Fig. 3H, Fig. S2B) of TNBC cell lines. In line, USP22 loss significantly reduced the CSC population of HCC1806 cells (Fig. S2C), strongly pointing to the CSC-supporting function of USP22 in this disease. Collectively, these data support the therapeutic value of USP22 and its role in promoting the CSC-based aggressive behavior of TNBC.

### USP22 sustains the mitochondrial biogenesis program, associated with a poor survival outcome in BLBC and HER2^+^-BC patients

**Fig. 4:**
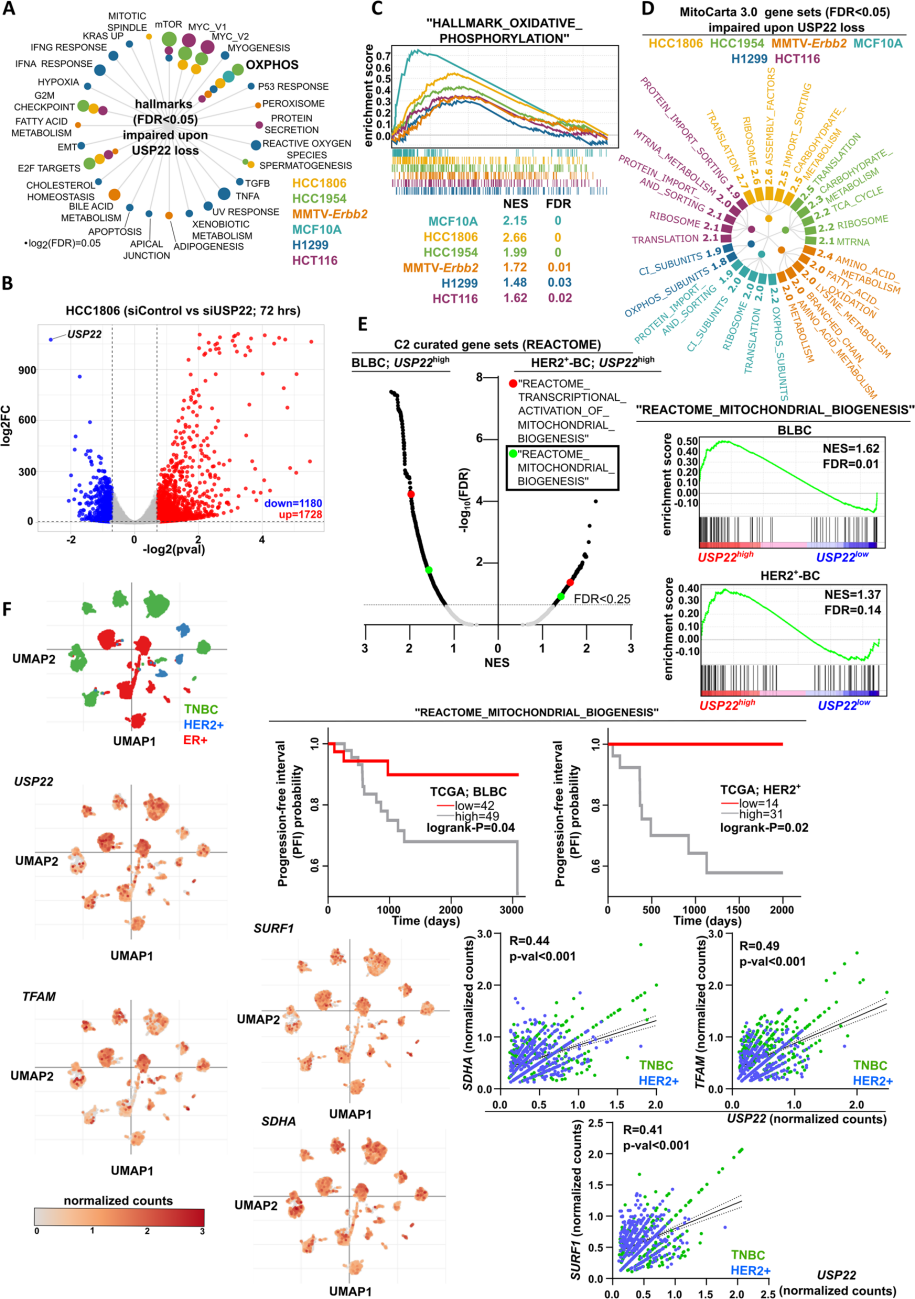
USP22 sustains the mitochondrial biogenesis program, associated with a poor survival outcome in BLBC and HER2^+^-BC patients: **A** Radar plot with all strongly enriched gene sets from the curated collection of "hallmark of cancer" gene sets indicates the "HALLMARK_OXIDATIVE_PHOSPHORYLATION" (OXPHOS) as the only commonly enriched gene set upon USP22 loss in all studied (non-)cancer models as well as in TNBC cells (accession number of all datasets is provided in Methods and Supplementary data). **B** Volcano plot of differentially expressed genes upon USP22 silencing in HCC1806 cells (TNBC) after 72 h of transfection (cut-off: |log2 fold change|≥ 0.7; *p*-val < 0.05 and basemean ≥ 15). **C** GSEA profiles of the "HALLMARK_OXIDATIVE_PHOSPHORYLATION" gene set enriched in all studied datasets with USP22 interference. NES: Normalized Enrichment Score. **D** GSEA of mitochondria-related gene sets in all studied USP22-deficient entities (gene set source: www.broadinstitute.org/mitocarta/mitocarta30-inventory-mammalian-mitochondrial-proteins-and-pathways). Values on the top of the bars represent the NES for each significantly enriched gene set. **E** Dot plot (upper left panel) of enriched gene sets from the curated collection of "REACTOME" gene sets in *USP22*^high^ BLBC and HER2^+^-BC patients (TCGA-BRCA) demonstrates two OXPHOS-related gene sets as significantly enriched in both cancer entities. The GSEA profile the "REACTOME_MITOCHONDRIAL_BIOGENESIS" is also shown in both cancer entities (upper right panel). Patient survival analysis robustly associates BLBC and HER2^+^-BC patients characterized by heightened expression of the "REACTOME_MITOCHONDRIAL_BIOGENESIS" gene set with a poor survival outcome (low panel). NES: Normalized enrichment score, source of patient transcriptomic and follow-up data: https://portal.gdc.cancer.gov/. **F** Uniform Manifold Approximation and Projection (UMAP) plots of *USP22*, *TFAM*, *SURF1* and *SDHA* expression in TNBC, HER2^+^ and ER^+^ BC biopsies at a single cell level. Correlation analysis of *USP22* with the same OXPHOS gene panel in TNBC and HER2^+^-BC biopsies at a single cell level (lower panel). Data were retrieved from https://singlecell.broadinstitute.org/single_cell

To characterize the USP22-dependent mechanisms supporting the CSC-phenotype in TNBC, we performed mRNA-sequencing (mRNA-seq) following USP22 silencing in HCC1806 cells. For this purpose, we proceeded to an unbiased approach by overlapping USP22-dependent transcriptomic programs identified in HCC1806 cells with several USP22 interference-based publicly available mRNA-seq datasets derived from the normal mammary epithelial cell line MCF10A, HER2^+^-BC models (HCC1954, MMTV-Erbb2), lung and colorectal cancer. Interestingly, our analysis revealed the "HALLMARK_OXIDATIVE_PHOSPHORYLATION" (OXPHOS) as the only commonly downregulated gene set across the different USP22-deficient systems (Fig. 4A-C). As the mitochondrial biogenesis program is indispensable for the CSC-based aggressive behavior in a wide range of cancer entities [8], we reasoned that USP22 may functionally support the mitochondrial homeostasis program. Indeed, by reperforming mitochondria-based pathway enrichment analysis using the MitoCarta v3.0 gene sets, we identified that in most of all studied transcriptomic datasets, mitochondrial import, ribosomal biogenesis, translation and respiratory chain assembly were substantially impaired upon USP22 loss (Fig. 4D). Concordantly, *USP22*^high^-BLBC and -HER2^+^-BC patients that showed a pronounced CSC signature (Fig. 2B-C) and poor survival outcome (Fig. 2D) display a concomitant enrichment for gene sets of the mitochondrial transcriptomic program (Fig. 4E, top panel). In line with these data, the same *USP22*^high^-patient cohorts characterized by an increased gene signature expression of the "REACTOME_MITOCHONDRIAL_BIOGENESIS" gene set demonstrate a significantly poorer survival outcome (Fig. 4E, bottom panel). In accordance, single-cell transcriptomic analysis of HER2^+^-BC and BLBC biopsies showed that *USP22* significantly correlates with the expression levels of several representative OXPHOS-related genes (Fig. 4F)*.* In conclusion, USP22 is markedly associated with the OXPHOS-transcriptomic program leading to poor survival outcomes in HER2^+^-BC and BLBC patients.

### USP22 sustains the OXPHOS-potential of HER2^+^-BC and TNBC cells

**Fig. 5:**
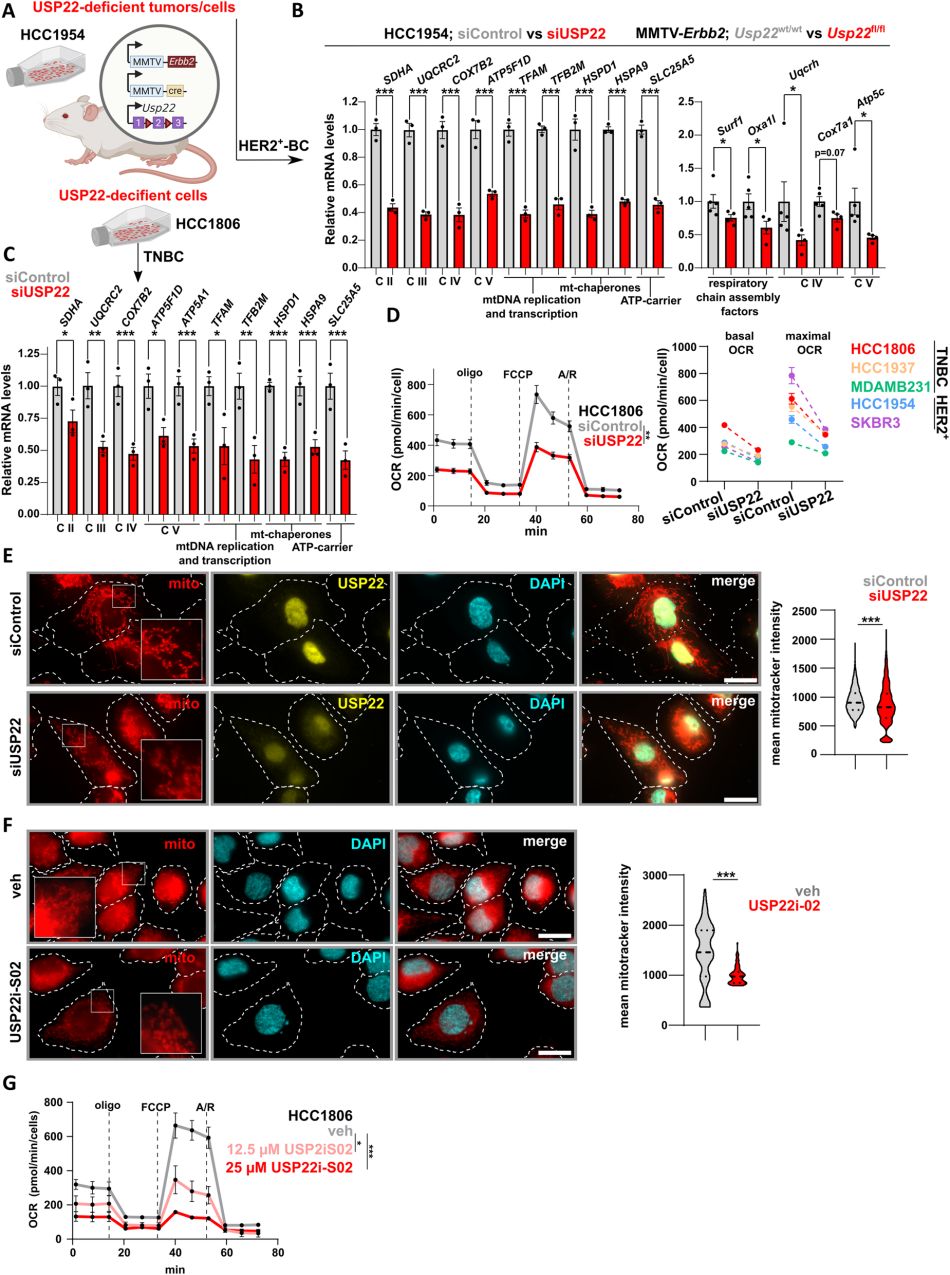
USP22 sustains the OXPHOS-potential of HER2^+^-BC and TNBC cells: **A** Schematic representation of the TNBC and HER2^+^-BC models utilized to study the dependency of OXPHOS on USP22. **B-E** Real-time quantitative PCR (RT-qPCR) (**B**-**C**), measurement of the oxygen consumption rate (OCR; **D**) and mitochondria staining using mitotracker (**E**) performed on TNBC and HER2^+^-BC models strongly present a downregulation of several OXPHOS-related genes, impaired OCR and altered mitochondria morphology upon USP22 depletion. E–F: White scale bar: 20 µm. **F-G** Mitotracker staining (**F**) and OCR measurement (**G**) in veh- and USP22i-S02-treated HCC1806 cells. Statistics: B, C: Student t-test; D, G: Student t-test (based on the area under the curve = AUC); **E**–**F** (right panel): non-parametric Mann–Whitney test. *p<0.05, **p<0.01, ***p<0.005. All experiments were performed in at least three biological replicates

Our findings revealed a robust link of USP22 to the OXPHOS program in HER2^+^-BC and BLBC patients. To confirm the USP22-dependent OXPHOS-transcriptomic program in these cancer entities, we performed quantitative real-time PCR (qRT-PCR). Here, USP22 loss significantly impaired the expression of several subunits of the respiratory chain (complex II-IV), ATP-synthase (complex V) and important mitochondrial chaperones and factors for mitochondrial DNA replication and transcription (Fig. 5A-C, Fig. S3A). Of note, we confirmed the same results by performing a single siRNA-based silencing of USP22, strongly arguing for the robustness of the used siUSP22 smart pool (Fig. S2D). Given the strong reduction of genes important for the OXPHOS machinery, we sought to investigate the respiratory potential of these cells. Indeed, USP22 loss significantly impaired the oxygen consumption rate (OCR) compared to their control counterparts (Fig. 5D, Fig. S3B). Consistently, immunofluorescence staining of mitochondria using mitotracker confirmed a decrease of mitochondrial abundance, length, perimeter and mean branch length in USP22-depleted TNBC cells (Fig. 5E, Fig. S3C). Consistently, USP22i-S02 similarly affected the overall mitochondrial amount and respiratory capacity of TNBC cells (Fig. 5F-G), arguing for a pharmacological means for interfering with the USP22-driven respiratory capacity in TNBC cells. Collectively, USP22 is indispensable for the mitochondrial biogenesis program in HER2^+^-BC and TNBC.

### The USP22-specific DUBm of the SAGA complex mediates the OXPHOS gene expression program and respiratory capacity in TNBC

**Fig. 6:**
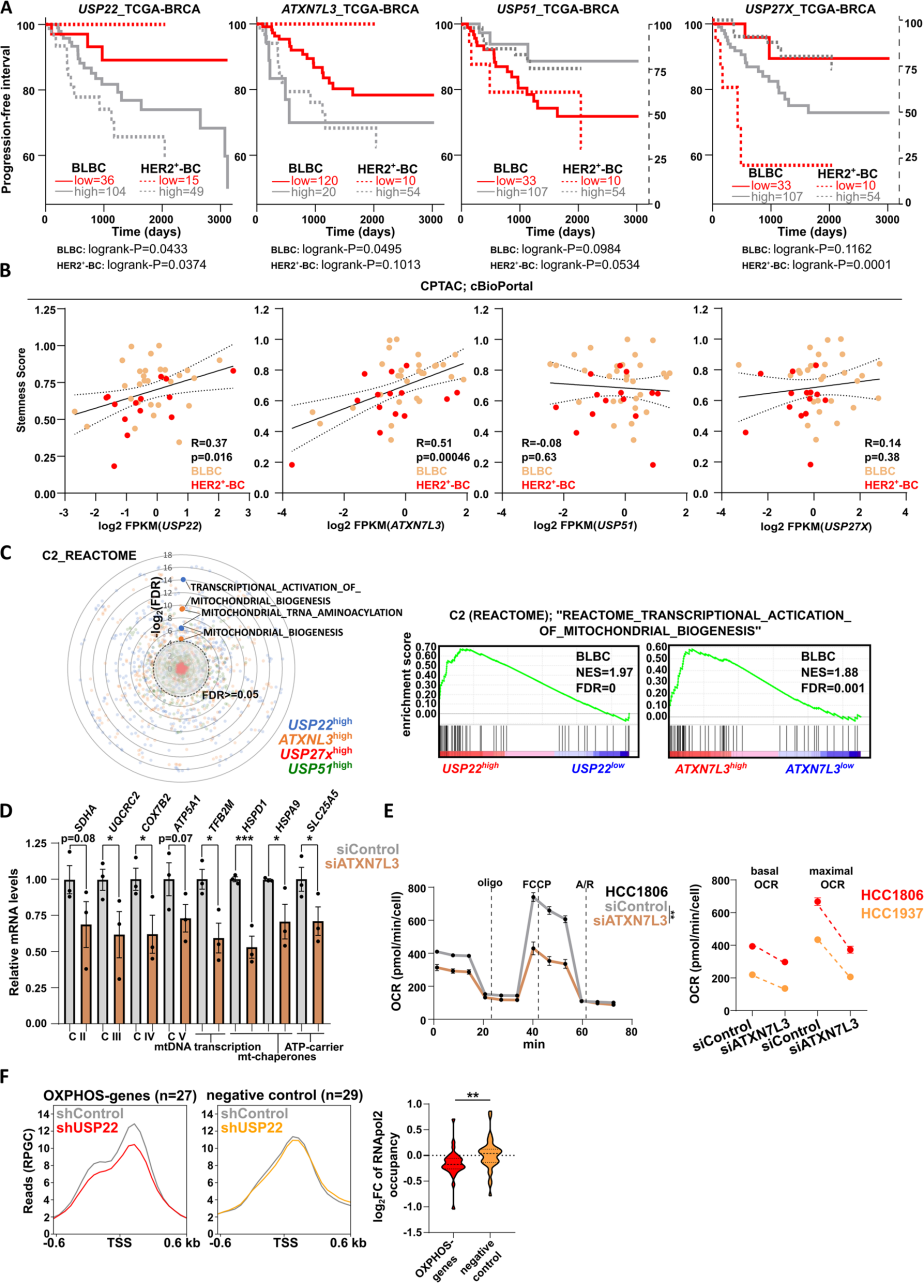
The USP22-specific DUBm of the SAGA complex mediates the OXPHOS gene expression program and respiratory capacity in TNBC:** A** Patient survival analysis show that in contrast to *USP51* and *USP27X*, *USP22* and *ATXN7L3* correlate with a poor survival outcome in BLBC and HER2^+^-BC patients (source: https://portal.gdc.cancer.gov/). **B** *USP22* and *ATXN7L3* RNA levels correlate with cancer stem cell characteristics BLBC and HER2^+^-BC biopsies (source: https://www.cbioportal.org/). **C** *USP22* and *ATXN7L3* expression is associated with several OXPHOS-related transcriptomic signatures, based on the C2_REACTOME curated gene set collection (left panel). Respective GSEA profiles of the "REACTOME_MITOCHONDRIAL_BIOGENESIS" gene set are also shown (right panel). **D-E** Loss of ATXN7L3 leads to an impaired expression of OXPHOS-related genes (**D**) and a drop of the OCR (**E**) in TNBC cell lines. **F** Aggregate profiles of RNA polymerase II (RNApol2) occupancy at the transcriptional start site (TSS) OXPHOS-related (left profile) and randomly selected genes (right profile) and the respective quantification of TSS-associated RNApol2 occupancy in shControl- and shUSP22-treated HCT116 cells (right panel; accession number: GSE121798). Statistics: Student t-test. *p<0.05, **p<0.01, ***p<0.005. **B**: Pearson correlation analysis; **C**, **D**-**F** (based on the area under the curve = AUC): Student t-test. NES: Normalized Enrichment Score. All experiments were performed in biological triplicates

As USP22 together with the USP22-accessory factors ATXN7L3 and ENY2, comprise the deubiquitinase module (DUBm) of the SAGA complex [24], we decided to ascertain the SAGA-dependent implication of USP22 in the OXPHOS-program. As expected, the DUBm-specific accessory protein Ataxin 7 Like 3 (*ATXN7L3*) was significantly correlated with a poor survival outcome (Fig. 6A) and stemness properties (Fig. 6B) in BLBC and HER2^+^-BC biopsies. In line, *ATXN7L3*^high^-expressing BLBC patients display a strong enrichment of the same OXPHOS-related gene sets as for *USP22*^high^-expressing BLBC patients (Fig. 6C). On the contrary, *USP51* and *USP27X *as alternative DUBm of the SAGA complex neither display a prognostic value (Fig. 6A-B) nor an association with any OXPHOS-related transcriptomic program in BLBC patients (Fig. 6C). Indeed, ATXN7L3-silenced TNBC cells showed a significant reduction of several OXPHOS-related genes (Fig. 6D), consequently leading to a loss of their respiratory capacity (Fig. 6E, Fig. S3D). Moreover, USP22 was identified as crucial during the earliest steps of transcription by licensing the formation of the transcription pre-initiation complex [25]. Therefore, we reasoned that USP22 loss would impact the RNA polymerase II (RNApol2) occupancy on the transcriptional start site (TSS), hence, the basal expression of OXPHOS genes. Indeed, downregulated OXPHOS genes showed a pronounced loss of occupancy of RNApol2 at their TSS (Fig. 6F) in USP22-deficient HCT116 cells. Collectively, USP22-specific DUBm of the SAGA complex mediates the OXPHOS gene expression program and respiratory capacity in BC cells.

### USP22 loss sensitizes BC cells to standard therapies and OXPHOS-specific inhibition

**Fig. 7:**
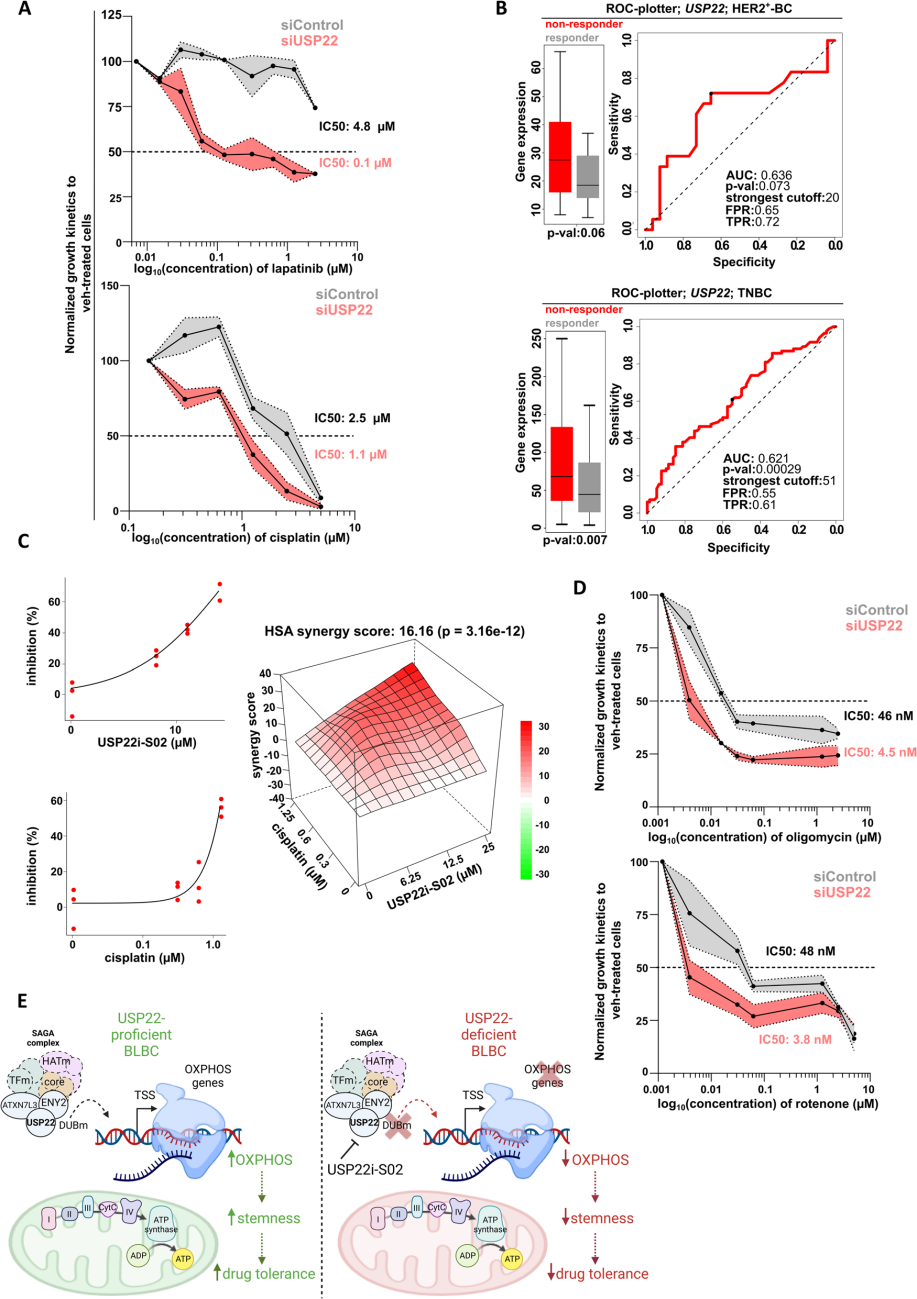
USP22 loss sensitizes BC cells to standard therapies and OXPHOS-specific inhibition: **A** Dose response assay of siControl- and siUSP22-treated MDAMB231 and HCC1954 cells with increasing doses of cisplatin and lapatinib, respectively. **B** Receiver operating characteristic (ROC) analysis indicates a poor response to standard therapies (left panel) and a high probability of disease recurrence (right panel) in BLBC and HER2^+^-BC patients with high expression of *USP22* (source: https://www.rocplot.org/). FPR: False Positive Rate, TPR: True Positive Rate. **C** A strong synergistic effect was observed in HCC1806 cells treated with increasing doses of USP22i-S02 and cisplatin. **D** Dose response assay of siControl- and siUSP22-treated MDAMB231 cells with increasing doses of rotenone and oligomycin. Statistics: **A**-**B** (left panel): Mann–Whitney test. All dose response experiments were performed in biological triplicates

USP22 was initially characterized as a member of an 11-gene "death-from-cancer" signature that strongly associated with a poor response to standard therapies and an overall decreased patient survival [13]. Moreover, USP22 has been broadly recognized as a potential therapeutic target for several malignancies and in particular, our group has recently demonstrated its promising therapeutic value in tackling the aggressive nature of HER2^+^-BC [16]. As expected, we confirmed that loss of USP22 impairs the expression of several chemotherapy resistance-associated genes (Fig. S3F) and sensitizes TNBC and HER2^+^-BC cells to increasing doses of cisplatin and lapatinib, respectively (Fig. 6A), pointing to an important role of USP22 in promoting drug-tolerance of BC cells. Consistent with these findings, using publicly available patient survival data we indeed identified that *USP22*^high^-expressing BLBC and HER2^+^-BC patients show a poor response to chemotherapeutics and anti-HER2 agents, respectively (Fig. 6B). In line, we identified that USP22i-S02 and cisplatin are synergistically exerting their antitumorigenic effect in HCC1806 cells, pointing to a novel USP22-based therapeutic scheme to optimize standard therapies in TNBC (Fig. 6C). Finally, we uncovered that USP22 loss sensitized TNBC cells to complex I and V inhibition by performing dose–response assays using rotenone and oligomycin, respectively (Fig. 6D), highlighting the clinical value of USP22 in optimizing OXPHOS-based therapeutic windows for this disease. In conclusion, USP22 strongly supports the drug-tolerant behavior of HER2^+^-BC and TNBC and is a promising therapeutic target to improve standard as well as OXPHOS-based therapies (Fig. 7E).

## Discussion

USP22 is a heavily studied DUB in cancer research due to its reported association with several aggressive features in many cancer entities [14]. Since its discovery as being strongly associated with poor patient survival in numerous malignancies, USP22 was reported to indeed possess a pro-tumorigenic function in various cancer models. Based on its deubiquitinating activity exerted on histone as well as non-histone targets, USP22 can influence many oncogenic signaling cascades [14]. In this work, we established for the first time that loss of USP22 interferes with the respiratory capacity of TNBC and HER2^+^-BC cells, ultimately impeding their tumorigenic and CSC properties. Cancer cell metabolism requires high energy levels and has the propensity to increase its glycolysis ratio, a metabolic state called aerobic glycolysis [26, 27]. On the other hand, an increasing number of studies started partially contrasting this broad cancer hallmark where OXPHOS activity is still maintained and is particularly essential for the energy supply of CSCs [28]. For instance, Gong et al. performed a metabolomic classification of 360 BLBC biopsies, demonstrating that OXPHOS activity is not only maintained but upregulated in cancer compared to the normal adjacent epithelium [29]. Furthermore, TNBC models harboring concomitant loss-of-function mutations of *TP53* and *RB1* (which account for up to 40% of TNBC cases; source: www.cbioportal.org; data not shown) demonstrated a particular vulnerability towards OXPHOS-specific inhibitors as quite efficient anti-CSC pharmacologic compounds [11]. In line with this, HER2^+^-BC cells and in vivo models exhibit a sustained therapeutic response to the respiratory complex I-specific inhibitor MitoTam that possesses anti-CSC properties and is currently undergoing phase I clinical trials [30, 31]. In this work, we demonstrated that siRNA-mediated USP22 depletion as well as using the USP22-specific small molecule inhibitor USP22i-S02 [32] impairs the OXPHOS-transcriptomic program and abrogates the basal respiratory capacity of HER2^+^-BC and TNBC cells. Importantly, the capacity of tumor cells to metabolically reprogram from aerobic glycolysis to sustained OXPHOS has been shown to strongly improve their drug-resistant traits [33] as well as sustain their anchorage-independent survival, ultimately facilitating metastasis [34]. In alignment, our findings showed that USP22-depletion dramatically enhanced cancer cell drug response to standard therapies as well as to complex I and complex V inhibitors. Given that several cancer entities become more dependent on OXPHOS following targeted therapies [35], USP22 should be considered a valuable therapeutic target to combat these aggressive properties in cancer patients. In conclusion, we posit USP22 as a major DUB in mastering the respiratory capacity and enhancing the drug-tolerant behavior of the two most particularly aggressive BC subtypes.

During the last years, several studies identified important pro-tumorigenic substrates stabilized by USP22 in BC. For example, an elegant study from Wang et al. showed that the estrogen receptor alpha (ERα) is deubiquitinated and stabilized by USP22, thereby promoting its transactivation function on estrogen-responsive gene targets and supporting the aggressive properties of ERα-positive BC [36]. Interestingly, a pioneer study from Kim et al. showed for the first time that USP22 promotes breast cancer by stabilizing the proto-oncogene c-Myc [37]. In accordance, Li et al. leveraged a chemotherapy-resistant in vitro TNBC model emphasizing the importance of the USP22/c-myc axis in maintaining the chemotherapy-resistant behavior of this malignancy [38]. Also, Gregory et al. confirmed an important role for USP22 in suppressing the neoantigen-specific immune response via stabilizing the ecto-5’-nucleotidase (NT5E) in BC [39]. To the best of our knowledge, so far, no previous study has reported the instrumental role of the USP22-DUBm/SAGA complex in sustaining the OXPHOS-transcriptomic program as a prerequisite to enhance the drug-tolerance of HER2^+^-BC and TNBC cells. Exceptionally, a recent study from Veronica De Luca et al. identified that the orthologues of USP22 and GCN5, namely Ubp8 and Gcn5l respectively, are indispensable for OXPHOS and that Gcn5l dictates the gene and protein expression of USP22 to sustain yeast cell respiration [40, 41]. However, while we examined the implication of the GCN5 acetyltransferase, as part of the histone acetyltransferase module (HATm) of the SAGA complex, in controlling cellular respiration, treatment with the GCN5-specific inhibitor MB-3 had no impact on the respiratory capacity of TNBC cells (data not shown). We posit this effect is presumably due to the redundant roles between human GCN5 and the homologous acetyltransferase p300-associated factor (PCAF) in gene activation [42] and the absence of a similar homologous counterpart in yeast. Although the latter yeast studies partially overlap with ours, supporting the functional role of USP22 in promoting the OXPHOS activity, our work reveals for the first time the pronounced therapeutic value of USP22 to combat certain OXPHOS-driven CSC-associated aggressive properties and improving response to standard therapies in HER2^+^-BC and BLBC.

An accumulating body of evidence suggests that USP22 is a remarkable DUB that can function as an important signaling hub for energy supply and angiogenesis as well as sustaining several energy-consuming anabolic processes in normal tissues and malignancies. For example, USP22 was reported to promote vascularization in the mouse placenta and aberrant angiogenesis in hepatocellular carcinoma and non-small cell lung cancer [43–45]. In addition, USP22 was shown to sustain the glycolytic capacity in BLBC and particularly under hypoxic conditions in hepatocellular carcinoma [38, 46]. Moreover, in the current work, we identified an important role of USP22 in promoting the mitochondrial biogenesis transcriptomic program, further strengthening the essential function of this DUB in the energy supply of cancer cells. Furthermore, our group has demonstrated the pivotal role of USP22 in fostering several energy-consuming cellular processes such as sustaining the gene expression of heat shock protein 90 alpha family class B member 1 to support the protein folding potential and survival of HER2^+^-BC cells [47]. Subsequently, an important work from our group showed that USP22 is indeed indispensable for the biology of HER2-driven mammary carcinoma in vitro and in vivo via deubiquitinating the heat shock protein 5 (HSPA5) chaperone, ultimately suppressing the pro-apoptotic axis of the unfolded protein response (UPR) [16]. Therefore, all the above-mentioned works combined place USP22 in the center of the energy-consuming protein folding and the energy and oxygen/nutrient supply processes to support cancer aggressiveness and poor response to standard therapies.

## Conclusion

The current work provides evidence that the USP22 deubiquitinase, part of the DUBm of the SAGA complex, supports the mitochondrial biogenesis transcriptomic program to foster the CSC traits and promote the drug resistant features of HER2^+^-BC and TNBC cells (Fig. 7). Concluding, we strongly consider USP22 as a promising therapeutic target to sensitize these aggressive malignancies to standard therapies and optimize patients' survival.

### Supplementary Information

**Additional file 1.**

## Data Availability

Publicly available data analyzed during the current study and their respective accession numbers are listed in Table S3 and accordingly mentioned where necessary in the figure legends. Raw sequencing data of HCC1806 treated with siUSP22 are accessible at ArrayExpress (E-MTAB-13577, https://www.ebi.ac.uk/arrayexpress/).
