# Supplementary material for: USP22 supports the aggressive behavior of basal-like breast cancer by stimulating cellular respiration
Source: Cell Commun Signal. 2024 Feb 12;22:120. doi: 10.1186/s12964-023-01441-5 (PMC10863169; doi:10.1186/s12964-023-01441-5)
Supplement: Supplementary file 2 — Additional file 1. [file 12964_2023_1441_MOESM1_ESM.docx]

**Supplementary Data**

**USP22 supports the aggressive behavior of basal-like breast cancer by stimulating cellular respiration**

Evangelos Prokakis^1,2,#^, Husam Bamahmoud^1^, Shaishavi Jansari^1^, Lena Fritsche^1^, Alexander Dietz^3^, Angela Boshnakovska^4^, Peter Rehling^4^, Steven A. Johnsen^2,5^, Julia Gallwas^1,^*, Florian Wegwitz^1,2, #,^*

*^1^Department of Gynecology and Obstetrics, University Medical Center Göttingen, Göttingen, Germany*

*^2^Department of General, Visceral & Pediatric Surgery, University Medical Center Göttingen, Göttingen, Germany*

*^3^Institute of Pathology, University Medical Center Göttingen, Göttingen, Germany*

*^4^Department of Cellular Biochemistry, University Medical Center Göttingen, Göttingen, Germany*

*^5^The Robert Bosch Center for Tumor Diseases, Stuttgart, Germany*

^#^Address correspondence to [eprokak@gwdg.de](mailto:eprokak@gwdg.de) (E.P) or [fwegwit@gwdg.de](mailto:fwegwit@gwdg.de) (F.W.)

* Equal author contribution

***Mammosphere assay***

Dissected mammary glands were pooled in 30 ml L15/10% FCS medium on ice. Tissues were minced using a gentleMACS™ Tissue Dissociators and gentleMACS™ M Tubes (Miltenyi Biotec) according to the manufacturer’s recommendation. Homogenates were transferred in a 50 ml falcon tube containing 35 ml collagenase/trypsin digestion mix in L15 medium (105 mg collagenase A, 52.5 mg trypsin, serum-free) and incubated on a horizontal shaker for 1 hour at 37 oC. Dissociated cells were centrifuged at 250 g for 5 min. Cell pellets were reserved. The fat layer and supernatant were centrifuged a second time at 250 g for 5 min. Fat layer and supernatant were discarded while pellets from both centrifugation steps were pooled and resuspended in 5 ml red blood lysis buffer (Sigma) for 5 min at room temperature. To remove fibroblast contamination, extracted primary cells were pelleted and resuspended in 10 ml DMEM/10% FCS medium and transferred to a T-80 tissue culture flask for 1 hour at 37 oC/5% CO2/5% O2. Flasks were gently shaken in a horizontal plane to ensure extracted organoids were in suspension and to be collected. The procedure was repeated one time with 5 ml L15/10% FCS medium and the supernatant was added to the previous one. Cell aggregates were centrifuged and resuspended in 1 ml 0.05% trypsin/EDTA for 5 min @ 37°C, centrifuged (250 g, 5min), resuspended in 2.5 ml L15/10% FCS with 5 μg/ml DNAseI for 5 min @ 37°C. DNA-digested samples were centrifuged and resuspended in 6 ml DMEM/F12 with B27 (Gibco, Thermofisher) and rEGF (20ng/ml). Afterward, the total amount of living cells was determined using trypan blue, and the cell suspension was diluted into 10.000 cells/ml in supplemented DMEM/F12. Finally, cells were seeded in a 96-well low adherent plate (150 cells/well) for spheroids, or in a 6-well adherent plate (150-300 cells/well) for colony formation. Mammary epithelial cells were cultured for at least 7 days and brightfield pictures were taken using a Celigo® S imaging cytometer (Nexcelom Bioscience LLC) when spheres were formed. Colonies were fixed with 100% methanol (10 min) and stained with 0.25% crystal violet in 20% methanol (for 20 min) when colonies were formed. Finally, stained colonies were scanned using EPSON perfection V700 PHOTO scanner.

***Functional assays***

All experiments were performed in biological triplicates. The results were plotted with GraphPad Prism v8.0.1

*Proliferation kinetics*: For the synergy assay with USP22i-S02 and cisplatin, 1000 cells per well were seeded in a 96-well plate. 24 hrs after seeding, treatment with the compounds was started for two cycles every 3 days. Confluency was recorded every 12 hrs over a period of 12 days using an IncuCyte® Live Cell Analysis System (Sartorius AG). For siControl-, siUSP22-, veh- and USP22i-S02-treated cells, 20.000 cells/well in a 24-well plate were reversely transfected using Dharmafect (based on manufacturer’s instructions) or treated with veh/USP22i-S02 after being adherent. Confluency was measured using a Celigo® S imaging cytometer (Nexcelom Bioscience LLC) for a time window until the control group reached confluency. Finally, cells were fixed with methanol for 10 min, stained with 1% crystal violet (Sigma) in 20% ethanol for 20 min and washed with tap water.

*Clonogenic assay*: 24 h post-transfection or for cells receiving DMSO or USP22i-S02, 1000 cells were seeded in a 6-well plate. 15 days after seeding, colonies were washed with PBS, fixed with methanol for 10 min, stained with 1% crystal violet in 20% ethanol for 20 min and washed with tap water. Treatment with inhibitor was performed every 5 days. Finally, stained colonies were scanned using EPSON perfection V700 PHOTO scanner. The number and size of colonies was assessed using ImageJ.

*Tumorsphere formation assay*: at 24 hours post-transfection or for cells receiving DMSO or USP22i-S02, 1000 cells per well were seeded in a low adherent 96-well plate. 15 days after seeding, spheres were photographed with a Celigo® S imaging cytometer (Nexcelom Bioscience LLC). Treatment with inhibitor was performed every 5 days. The number and size of spheres was assessed using ImageJ.

*Gap closure assay*: at 48 hours post-transfection or for cells receiving DMSO or USP22i-S02, 70000-90000 cells per silicon chamber side were seeded. Silicon chamber was removed until cells were adherent, washed twice with PBS and replaced with appropriate medium. Inhibition treatment was performed once without washing out until gaps were closed.

**Imaging and analysis workflow of DAB-stained USP22 in breast biopsies**

Pictures were acquired in a TIFF format with an IX-83 Olympus Microscope device. Pictures were opened in ImageJ (v1.54g) and converted to RGB format. Subsequent color deconvolution of H (hematoxylin) and D (DAB). As USP22 was in general expressed in all stained tissues, DAB was used as a region of interest (ROI) chanel to mark the nuclei. For this purpose, a copy of the DAB channel was thresholded (upper=1e330, lower=185), then a binary processing was applied (median with rad=1 and pressing the watershed option). Thereafter, number of particles was retrieved using the following criteria: size:55-300, circularity:0.7-1.0 and mean and area was clicked. Finally the ROI overlay was transferred to the other copy of the DAB channel which was in advance inverted and mean DAB intensity in the ROI was obtained. DAB mean values were subsequently graphed using Graphpad Prism (v.8.0.1).

**Protein isolation and western blot analyses**

For protein isolation from siRNA-trasfected cells without (total 48 hours of transfection) or with inhibitor treatment [total 72 hours of transfection with 1.25 μM of dorsomorphin (Selleckhem) for the last 24 hours], 300 000 cells per well in a 6-well plate were seeded.of Radioimmunoprecipitation Assay Buffer (RIPA; 10 mM Tris-Cl pH 8, 1 mM EDTA, 1% v/v Triton X-100, 0.1% sodium deoxycholate, 0.1% SDS, 140 mM NaCl) supplemented with protease and phosphatase inhibitors was used (1 μM activated orthovanadate, 10 mM β-glycerophosphate disodium salt hydrate, 10 mM Pefablock, 10 mM N-Ethylmaleimide, 1 mM Aprotinin/Leupeptinin, 1mM NaF, 1 μM iodoacetic acid). Cells were washed once with PBS and 200 μl of RIPA buffer was added to each well (6 well plate). After 10 min incubation on ice, cells were scraped and lysates were sonicated for three cycles, 5 min each using a Bioruptor (Diagenode). Laemmli buffer (375 mM Tris/HCl, 10% SDS, 30% glycerol, 0.02% bromophenol blue, 9.3% DTT) was added to each lysate and cooked at 95°C for 5 min before protein separation with a 10 to 12% polyacrylamide gel. Proteins were transferred to nitrocellulose membrane (0.45 µm pore, Immobilon, Millipore), blocked with 5% skimmed milk in TBS-T for 1 hour and incubated with primary antibody overnight at 4°C. The day after, the membrane was washed with TBS-T, incubated 1 h with secondary antibody at room temperature. After a final wash step, protein detection was achieved with the Millipore substrate in an Intas Chemostar Imager (Intas Science Imaging). Used primary antibodies are listed in Table 4.

***RNA isolation and real-time quantitative PCR (RT-qPCR)***

For RNA isolation from siRNA-trasfected cells (total 48 hours of transfection) without or with inhibitor [total 72 hours of transfection with 10 μM of TRULI (MedChemoExpress) for the last 48 hours), 120 000 cells per well in a 12-well plate were seeded. For RNA isolation from BAY-1251152 [CDK9i ( at 250 nM, 6 and 48 hours of treatment)-treated cells, 150.000 cells per well in a 12-well plate were seeded. At the end of treatment, cells were washed with PBS and lysed in 500 µl EXTRAzol (EM30-100). Lysates were then collected and RNA was extracted, as previously described (1,2). Reverse transcription of 1 µg RNA was performed using M-MuLV reverse transcriptase (NEB) with random primers according to the manufacturer’s instructions. The expression of specific genes was finally estimated by quantitative real-time PCR using a CFX Connect™ Real-Time System (Bio-Rad). Gene expression levels were normalized relative to the *RPLP0* housekeeping gene (all RT-qPCR experiments) or *RNA18S5* (Figure 6J). RT-PCR program: 1x 2 min-95 ^o^C, 40x 10 sec-95 ^o^C followed by 1x 30 sec-60°C. Primers (Table S2) were designed using the online tool https://www.ncbi.nlm.nih.gov/tools/primer-blast/ and were ordered from Sigma-Aldrich (Germany). The results were finally plotted with Graphpad Prism v8.0.1.

***Supplementary figure legends***

**Fig.S1: A** RT-qPCR of USP22 in siControl- and single siUSP22- and smart pool USP22 siRNA-treated HCC1806 cells. **B-D** Growth kinetics (B), colony formation (C) and sphere formation assay (D) in siControl-and siUSP22-treated BLBC cells. **E-G** Growth kinetics (E, 12.5 µM), colony formation (F, MDAMB231: 25 µM, HCC1937: 50 µM) and sphere formation assay (G, 50 µM) in veh- and USP22i-S02-treate BLBC cells. Statistics: A: Student t-test; B,E (based on the area under the curve; AUC), C-D, F-G (right panel): Student t-test; C-D, F-G (right panel): Mann-Whitney test. *p<0.05, **p<0.01, ***p<0.005. All experiments were performed in biological triplicates.

**Fig.S2:** **A-B** Gap closure assay in siControl- or siUSP22-treated (A) or DMSO- (veh) or USP22i-S02-treated (B, HCC1937: 50 µM, MDAMB231 and MDAMB468: 25 µM) BLBC cells. **C** FACS-based CD44 and CD24 staining in siControl- and siUSP22-treated HCC1806 cells. **D** RT-qPCR or several OXPHOS-related genes in siControl- and single USP22-specific siRNAs-treated HCC1806 cells. Statistics: A-B (based on the area under the curve; AUC), C (lower panel), D: Student t-test. *p<0.05, **p<0.01, ***p<0.005. All experiments were performed in biological triplicates.

**Fig.S3: A** RT-qPCR of several OXPHOS genes in siControl- and siUSP22-treated MDAMB231 and HCC1937 cells. **B** OCR measurement in siControl- and siUSP22-treated TNBC and HER2^+^-BC cells. **C** Violin plot of the median mitochondrial perimeter, area and branch length in siControl- and siUSP22-treated HCC1806 cells. **D** OCR measurement in siControl- and siATXN7L3-treated HCC1937 cells. **E** RT-qPCR of drug resistance genes (*ABCB1*, *ABCG2*, *ALDH1A3*) in siControl- and siUSP22-treated HCC1806 cells. Statistics: A, B, D, (based on the area under the curve; AUC) and E: Student t-test, C: non-parametric Mann-Whitney test. *p<0.05, **p<0.01, ***p<0.005. All experiments were performed in biological triplicates.

***Supplementary Tables***

**Table S1: siRNAs used in this study**.

| **Gene** | **siRNA** | **Cat. Number (Dharmacon)** |
| --- | --- | --- |
| Non-Τargeting #5 | UGGUUUACAUGUCGACUAA | D-001210-05-20 |
| *USP22* | #1 GGAGAAAGAUCACCUCGAA | D-006072-01 |
|  | #2 CAAAGCAGCUCACUAUGAA | D-006072-02 |
|  | #3 GGAAGAUCACCACGUAUGU | D-006072-04 |
|  | #4 CCUUUAGUCUCAAGAGCGA | D-006072-17 |

**Table S2: RT-PCR primers used in this study**.

| **Gene name** | **Forward (5‘-3‘)** | **Reverse (5‘-3‘)** | **Species** | **Reference** |
| --- | --- | --- | --- | --- |
| ***RPLP0*** | GATTGGCTACCCAACTGTTG | CAGGGGCAGCAGCCACAAA | Human | (1) |
| ***USP22*** | AGCCAAGGGTGTTGGTCGCG | ACTGCCACCACGCCCGAAAG | Human | (1) |
| ***SDHA*** | TGGAGATCCGAGAAGGAAGA | AGCGAAGATCATGGCTGTCT | Human | This study |
| ***UQCRC2*** | TGGAATTGAAGCAGTTGGTG | GGTGCTGTGGTGACATTGAG | Human | This study |
| ***COX7B2*** | AAGGTTCGGAATTTGCCTCT | AGGATTGCAGTTGCCTTCAG | Human | This study |
| ***ATP5F1D*** | ACTCTTCGGTGCAGTTGTTG | ATTCGGATCTGGATCTCTGC | Human | This study |
| ***TFAM*** | TCACAATGGATAGGCACAGG | GCAGAAGTCCATGAGCTGAA | Human | This study |
| ***TFB2M*** | GATCGGAGATTGGCTGAGAC | CTTTGGCACCAGCTTCAAGT | Human | This study |
| ***HSPD1*** | TTGGGGAAGTCCCAAAGTAA | TCCCCAGCTTCTTCATTTGT | Human | This study |
| ***HSPA9*** | CCTTGATGTCACTCCCCTGT | ATTTCCACTTGCGTTTGACC | Human | This study |
| ***SLC25A5*** | CACCCAGGCTCTTAACTTCG | CACAAAACACAGGGATGTGG | Human | This study |
| ***Surf1*** | CTGTCAGGAGGAGCGTCTTT | AGCAGGAACCACTGGAGAAA | Mouse | This study |
| ***Oxa1l*** | CGAATGCAGAAACACTTGGA | GATGCTGCTGTTGTTGCTGT | Mouse | This study |
| ***Uqcrc2*** | TACTCTGGTTGCGCTTGTTG | CACTGTTGTTAGGGGGTCCA | Mouse | This study |
| ***Cox7a1*** | TTAGAAAACCGTGTGGCAGA | CAGCGTCATGGTCAGTCTGT | Mouse | This study |
| ***Atp5c*** | CAGCAGCTGGGAAAGAAGTT | TCCGTCCCACATCTTTGAAT | Mouse | This study |
| ***ABCB1*** | AGGCCAACATACATGCCTTC | AGCTATGGCAATGCGTTGTT | Human | This study |
| ***ABCG2*** | GCAAATGCTGTCCTTTTGCT | GGCCAATAAGGTGAGGCTATC | Human | This study |
| ***ALDH1A3*** | CCGACTATGGACTCACAGCA | TGTGCATAGAGGGCGTTGTA | Human | This study |

**Table S3: mRNA- and ChIP-seq accession numbers used in this study**

| **Tissue** | **Dataset** | **Accession** |
| --- | --- | --- |
| HCC1806 | **RNA sequencing** | E-MTAB-13577 |
| MCF10A | **RNA sequencing** | E-MTAB-8247 |
| HCT116 | **RNA sequencing** | E-MTAB-7393 |
| MMTV-*Erbb2* | **RNA sequencing** | E-MTAB-9331 |
| HCC1954 | **RNA sequencing** | E-MTAB-8256 |
| H1299 | **RNA sequencing** | GSE131934 |
| HCT116 | **ChIP sequencing** | GSE121798 |

**Table S4: List of primary antibodies**.

|  | **WB** | **IHC** | **FACS** | **cat.number, company** |
| --- | --- | --- | --- | --- |
| **ACTIN** | 1:200 |  |  | sc-1616(1-19)  Santa Cruz |
| **USP22** | 1:1000 | 1:100  (citric buffer, pH:6) |  | HPA044980  Sigma Aldrich |
| **H2Bub1** |  | 1:50 |  | Home-made |
| **H2B** | 1:1000 |  |  | Abcam  ab1790 |
| **anti-CD44-APC, im7** |  |  | 1:500 | 103011  biolegend |
| **anti-CD24-FITC, ML5** |  |  | 1:500 | 311103  biolegend |

***Supplementary References***

1. Prokakis E, Dyas A, Grün R, Fritzsche S, Bedi U, Kazerouni Z, et al. USP22 promotes HER2-driven mammary carcinoma aggressiveness by suppressing the unfolded protein response. Oncogene. 2021 Jun;40(23):4004–18.
